# The Daily Mile makes primary school children more active, less sedentary and improves their fitness and body composition: a quasi-experimental pilot study

**DOI:** 10.1186/s12916-018-1049-z

**Published:** 2018-05-10

**Authors:** Ross A. Chesham, Josephine N. Booth, Emma L. Sweeney, Gemma C. Ryde, Trish Gorely, Naomi E. Brooks, Colin N. Moran

**Affiliations:** 1 Faculty of Health Sciences and Sport, University of Stirling, Scotland, FK9 4LA UK; 2 Institute of Education, Community and Society, Moray House School of Education, University of Edinburgh, Scotland, EH8 8AQ UK; 3 Present address: School of Health, Social Care and Life Sciences, University of the Highlands and Islands, Centre for Health Sciences, Old Perth Road, Inverness, IV2 3JH UK

**Keywords:** Daily Mile, Children, Physical activity, Primary school, Fitness, Body composition

## Abstract

**Background:**

The Daily Mile is a physical activity programme made popular by a school in Stirling, Scotland. It is promoted by the Scottish Government and is growing in popularity nationally and internationally. The aim is that each day, during class time, pupils run or walk outside for 15 min (~1 mile) at a self-selected pace. It is anecdotally reported to have a number of physiological benefits including increased physical activity, reduced sedentary behaviour, increased fitness and improved body composition. This study aimed to investigate these reports.

**Methods:**

We conducted a quasi-experimental repeated measures pilot study in two primary schools in the Stirling Council area: one school with, and one without, intention to introduce the Daily Mile. Pupils at the control school followed their usual curriculum. Of the 504 children attending the schools, 391 children in primary classes 1–7 (age 4–12 years) at the baseline assessment took part. The follow-up assessment was in the same academic year. Outcomes were accelerometer-assessed average daily moderate to vigorous intensity physical activity (MVPA) and average daily sedentary behaviour, 20-m shuttle run fitness test performance and adiposity assessed by the sum of skinfolds at four sites. Valid data at both time points were collected for 118, 118, 357 and 327 children, respectively, for each outcome.

**Results:**

After correction for age and gender, significant improvements were observed in the intervention school relative to the control school for MVPA, sedentary time, fitness and body composition. For MVPA, a relative increase of 9.1 min per day (95% confidence interval or 95%CI 5.1–13.2 min, standardised mean difference SMD = 0.407, *p* = 0.027) was observed. For sedentary time, there was a relative decrease of 18.2 min per day (10.7–25.7 min, SMD = 0.437, *p* = 0.017). For the shuttle run, there was a relative increase of 39.1 m (21.9–56.3, SMD = 0.236, *p* = 0.037). For the skinfolds, there was a relative decrease of 1.4 mm (0.8–2.0 mm, SMD = 0.246, *p* = 0.036). Similar results were obtained when a correction for socioeconomic groupings was included.

**Conclusions:**

The findings show that in primary school children, the Daily Mile intervention is effective at increasing levels of MVPA, reducing sedentary time, increasing physical fitness and improving body composition. These findings have relevance for teachers, policymakers, public health practitioners, and health researchers.

**Electronic supplementary material:**

The online version of this article (10.1186/s12916-018-1049-z) contains supplementary material, which is available to authorized users.

## Background

The Daily Mile is a physical activity intervention developed at St Ninian’s Primary School (Stirling, Scotland) in 2012 [1]. The initial aim was to improve the fitness of children; although, there have since been many additional benefits anecdotally reported by children, teachers and parents [1]. These include improved physical activity, sedentary time, physical fitness, body composition, sleep, diet, concentration, well-being and obesity levels. However, no objectively measured scientific evidence has yet been gathered on the validity of these reports.

The successful implementation of the Daily Mile at many schools, its continued maintenance and its increasing popularity seem to be a result of the simplicity of the activity, the autonomy given to classroom teachers over when they do it during the school day and the pupil-determined pace. Since its development, the Daily Mile has been rolled out across the country by the Scottish government [2]. It is estimated by Education Scotland that, from local authorities who responded to their query, ~ 50% of primary schools in Scotland are already doing the Daily Mile with a further 18% planning to do the Daily Mile soon (personal communication). It has also become popular in the rest of the UK with interest from the UK government [3, 4]. Additionally, it has been introduced in the Netherlands, Belgium and parts of the USA with interest from many other countries [5], despite the lack of rigorous evidence on the efficacy of taking part.

Globally, physical activity levels are low [6]. Furthermore, low physical activity levels in childhood are predictive of low physical activity levels in adulthood [7]. The World Health Organisation (WHO) considers policies and interventions to increase physical activity levels to be important in all age groups and that any potential harm of increasing physical activity is outweighed by the associated benefits [8, 9]. In a recent accelerometer study on children (2–11 years old) from eight European countries, the proportion of children achieving 60+ minutes per day (recommended for 5–18 year olds [10]) of moderate to vigorous physical activity (MVPA) ranged from 9.5% to 34.1% in boys and from 2.0% to 14.7% in girls [11]. MVPA levels generally decline with age in both genders as sedentary time increases [12]. MVPA levels are also believed to be influenced by socioeconomic status; however, systematic reviews are unclear on the consistency of this relationship and more evidence needs to be gathered [13]. School-based physical activity interventions like the Daily Mile are appealing because they include whole classes, therefore they reach many children regardless of socioeconomic status, physical activity level or fitness level. They also break up sedentary time as they occur during lessons and have the potential to reach much larger proportions of the population than opt-in groups like sports clubs. Therefore, understanding the impact of the Daily Mile on physical activity and sedentary behaviour levels is of key importance.

Overweight and obesity rates are of pandemic proportions and considered to be a key target for the WHO [8]. In Scotland, 30% of children (29% of boys and 32% of girls) aged 7–11 years were overweight or obese in 2015 [14], a figure similar to that in England [15]. At the same time, there is evidence of a decline in the performance of children in the 20-m shuttle run (an indicator of physical endurance fitness) [16]. Low fitness and low levels of physical activity in adults and children are associated with a number of risk factors for non-communicable diseases and adverse health outcomes including obesity, cardiovascular disease, diabetes, some cancers, low mood and poor cognitive function [17–21]. Additionally, both overweight and low fitness are also known to be related to lower socioeconomic status [22, 23]. Studies into the impact of the Daily Mile on fitness and body composition are important for understanding its potential to improve public health, including health inequalities, and for developing future public health policies.

The Daily Mile at least has the potential to impact on key areas of global public health. Whilst published evidence shows a positive health relationship between physical activity, particularly MVPA, and these outcomes [24], it is unknown whether 15 min of exercise, particularly with no expectation of intensity, will provide such benefits. Given the associated loss of academic classroom time (up to 75 min per week), it is paramount that evidence on the impact of the Daily Mile on each anecdotally reported benefit is gathered to ensure that government policies are appropriate. Furthermore, any associated benefits in physiological health are likely to be small [25]; thus, it is essential to use gold standard measurement techniques. Therefore, the aims of this study are to assess the anecdotally reported physiological benefits of participation in the Daily Mile. Specifically, using a repeated measures design and gold standard measurement techniques, we will assess whether the introduction of the Daily Mile into a primary school setting leads to increased MVPA, reduced sedentary time, improved fitness and improved body composition.

## Methods

### Study design and ethics

“Using the Daily Mile to turn the WHEEL” (Well-being, Health, Exercise, Enjoyment and Learning) is a school-based quasi-experimental study designed to assess the anecdotally reported benefits of taking part in the Daily Mile. Ethical approval was obtained from the University of Stirling, School of Sport Research Ethics Committee (reference number 760). Approval was also obtained from the Director of Children, Young People and Education at Stirling Council.

### Eligibility and recruitment

**Fig. 1:**
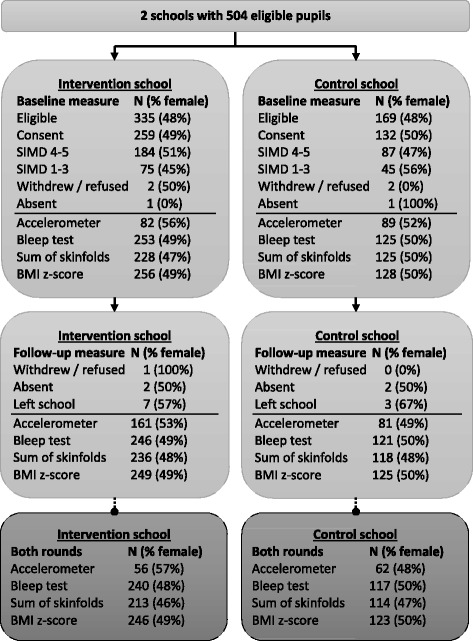
Trial profile. *N* is the number of school pupil participants. The percentage of female participants for each measurement is shown in parentheses. Individual boxes show the totals for each school at baseline, or at follow-up, or the totals for participants with valid measurements in both rounds. Not all pupils with follow-up measurements had been measured at the baseline (or vice versa), since different pupils were absent at the main and follow-up assessments, some refused to complete certain measurements at each time point and some pupils left or moved between schools. BMI body mass index, SIMD Scottish Index of Multiple Deprivation

State primary schools in the Stirling Council areas were eligible for inclusion. Recruiting from only one council area reduced any potential variance in the delivery of education that may have impacted on outcome measurements. Two local primary schools were identified and approached: one that was not doing the Daily Mile but was intending to introduce it (the intervention school) and one that was not doing the Daily Mile and did not intend to introduce it (the control school). Both schools had expressed a desire to introduce the Daily Mile although the control school felt that it would not be possible due to the layout of its playground. Both schools had a range of levels of deprivation, although the majority of pupils were from higher socioeconomic quintiles (Fig. 1). Participants were children in all years (age 4–12 years) at the time of recruitment.

Once the schools had agreed to participate in the study, parents and guardians of the children were sent a letter and information sheet about the study with an opt-in consent form. For children in primary classes 4–7 (age 7–12 years), an additional child consent form was included. Information sessions were held in both schools to allow parents to ask questions and see the study equipment. They were also given the opportunity to contact the research team by email or phone to discuss the study. Information about being able to withdraw from the study at any stage was given in the information sheet, consent form and verbally. All of the children were also asked to confirm verbally that they were happy to take part on each day of testing.

### Intervention

The Daily Mile is a school-based physical activity intervention made popular by a primary school in the Stirling Council area, Scotland [1]. It involves children going outside, at a time of the classroom teacher’s choosing, for ~ 15 min of exercise at a pace self-selected by each individual child. This is done during normal classroom time and is in addition to time spent in physical education or scheduled breaks. Typically, it involves laps of a football pitch or playground area. Children often talk as they go and perform a mixture of walking and running. Those who run the whole time will complete ~1 mile in 15 min. Children wear their normal school clothes; most wear their normal school shoes and jackets are only worn in cold or wet weather. It is completed on most days regardless of weather conditions. A leaflet produced by the originator school was given to the school implementing the Daily Mile. No additional instructions for initiation of the Daily Mile were given by the research team.

### Participant involvement

The outcome measures selected for the study were chosen based on anecdotal reports of the influence of taking part in the Daily Mile. This information had been gathered from the children, their parents and their teachers by the originator school. The research question, study design and specific outcome measures were developed in part during meetings with the head teacher at the originator school, who was familiar with this information. After publication, the results from this study will be disseminated to the schools involved as copies of the manuscript and infographics, and during question and answer sessions in the schools.

### Outcome measures

The primary physiology-related outcome measurements for the WHEEL project were accelerometer-assessed MVPA and sedentary time, fitness assessed using a 20-m shuttle run, and body composition assessed using skinfolds. Additional, cognitive- and well-being-related outcome measurements will be reported elsewhere.

### Participant assessments

Baseline assessments (before the intervention) were carried out in October 2015 for the intervention school and March 2016 for the control school. Outcome assessments were completed in May 2016 for the intervention school and June 2016 for the control school. Identical protocols and procedures were used at both assessments. They were undertaken by trained fieldworkers. The fieldworkers worked in groups such that at least one member of each group was disclosure-checked under the Protecting Vulnerable Groups scheme [26]. Children completed tasks in a random order and were given stickers for completing each measurement type. Testing sessions lasted between 1 and 2 h and were carried out over 2 weeks, depending on class size and school timetable.

ActiGraph accelerometers were used to assess physical activity and sedentary time. Five models of accelerometer were used: ActiGraph wGT3X-BT, wGT3X+, GT3X+, GT3X and GT1M. The GT1M and GT3X monitors are comparable when classifying total time spent in specific intensity categories [27]. There is also strong agreement between the GT1M, GT3X and GT3X+ accelerometers, making it acceptable to use them in the same study [28]. The primary difference between the wGT3X-BT and GT3X+ accelerometers is the ability of the wGT3X-BT to communicate wirelessly and all measurements should function identically (personal communication from ActiGraph). Accelerometers were worn around the waist on the left side with the same orientation to standardise the position. Children were asked to wear the belt for eight consecutive days during waking hours (except when bathing or swimming). A poster and instruction sheet with visual and written prompts were provided to each child to remind them of how and when to wear the accelerometer. Upon collection by the research team, the data were downloaded with the ActiLife 6 software (ActiGraph LLC, USA). A valid measurement required at least 10 h wear for 3 days [29]. A 60-s epoch was used and non-wear time was defined as strings of consecutive zeros lasting 60 min or more [30]. The accelerometer output is in counts per minute (cpm). Evenson cut points [31] were used to define time spent being sedentary (≤100 cpm) and time spent in MVPA (≥2296 cpm). The accelerometer data were corrected for wear time in addition to gender and age in days on the day of testing in the main analysis.

The maximal multistage 20-m shuttle run test [32] was assessed using the Multistage Fitness Test CD (Sports Coach UK, UK) using the standard procedure. Cones marked shuttle boundaries and lanes. Between four (younger) and eight (older) children completed the test at the same time depending on their age group. Instructions and a demonstration of the test were provided prior to each group participating and additional verbal instructions were provided during the test as necessary. The test began at 8.5 km·h^− 1^ and after each minute increased by 0.5 km·h^− 1^. When a child was unable to reach the 20-m line prior to the bleep twice in a row, they were asked to stop and their level and shuttle score was recorded. Shuttle run tests were performed outside on tarmac. Age-corrected $\dot{\mathrm{V}}{\mathrm{O}}_2\max$ scores were created according to the method of [32] for comparison with other studies (Additional file 1: Tables S1–S3). However, this corrects for age in years and therefore, it was not used for the main analysis. Instead, shuttle distance corrected for gender and age in days on day of testing was used to give an improved resolution to the correction in the main analysis.

All skinfold measurements were completed with the child behind a privacy screen with at least two disclosure-checked fieldworkers present. Fieldworkers taking skinfold measurements were trained by an International Society for the Advancement of Kinanthropometry (ISAK) Level 3 instructor prior to involvement in the study and all measurements were taken according to standard ISAK procedures [33]. Triceps, biceps, iliac crest and subscapular skinfolds were measured using Harpenden skinfold callipers (Baty International, UK). All measurements were taken from the right-hand side with the child standing. Where appropriate, they stood on an anthropometric box. Skinfold measurements were summed prior to analysis to give a sum of skinfolds (in millimetres). Skinfold data were corrected for gender and age in days on day of testing in the main analysis.

Height was measured, to the nearest 1 mm, without shoes using the Leicester Height Measure (Seca, UK) according to standard ISAK procedures [33]. Body weight was measured without shoes in light clothing to the nearest 0.1 kg using electronic Sensa 804 scales (Seca, UK). Height (m) and weight (kg) were used to calculate the body mass index (BMI = weight/height^2^). BMI *z* scores relative for age were calculated using UK 1990 reference data in the LMS Growth add-in for Microsoft Excel [34]. Healthy weight was defined as BMI *z* score < 1.04, overweight as BMI *z* score of 1.04–1.63 and obesity as BMI *z* score ≥ 1.64.

All anthropometric measurements were taken twice and the average taken for the analyses. Where there was a substantial difference between the two measurements, a third measurement was taken and the median value was used for the analyses.

### Additional Information

Schools provided the date of birth and postcodes for all consented pupils. The date of birth allowed the analyses to be corrected for age on day of testing. The postcode allowed the assignment of the Scottish Index of Multiple Deprivation (SIMD) [35]. SIMD combines data from seven different domains of deprivation into a single score: income, employment, health, education, access to services, crime and housing. However, it should be noted that this gives a postcode-specific deprivation score that may not reflect that of an individual household.

### Statistical analyses

Descriptive statistics, Pearson χ^2^ and odds ratios were calculated in Excel 2013. Baseline group frequency comparisons were performed using a Pearson χ^2^ test. Baseline group mean comparisons were performed using Student’s *t* test (uncorrected) or general linear model ANOVA (corrected for age at time of testing, gender and age*gender). The main analyses were performed in SPSS Statistics (version 21.0.0.1). General linear model regression analyses with repeated measures were used to investigate the effect of doing the Daily Mile. Analyses of all outcome measures included an adjustment for the common confounders: age at time of testing, gender and age*gender. This controls for the effects of age and gender and any different effects of age in the two genders as well as any differences in length of time in the study. Analyses for MVPA and sedentary time were additionally corrected for accelerometer wear time as a covariate. Analyses were conducted first without and then with a correction for SIMD. Standardised mean differences (SMDs), or effect sizes, are calculated as a change measured in the intervention school relative to the control school as a proportion of the pooled standard deviation of the change. An SMD of 0.2–0.5 is considered to be small, an SMD of 0.5–0.8 is considered to be medium and an SMD of 0.8 or above is considered to be large [36]. No correction for multiple testing was made since all four primary outcome measures were anecdotally reported to be influenced by the Daily Mile.

## Results

Figure 1 shows the study profile. Overall 77.6% of eligible pupils consented to the study (77.3% in the intervention school and 78.1% in the control school). A total of 371 pupils (247 and 124 in the intervention and control schools respectively) provided at least one measurement in both rounds of testing. All consented children, irrespective of whether they had all measurements or not, are included in Fig. 1. For each outcome measurement, the information in Tables 1 and 2 is based only on children who had measurements at both time points.

**Table 1:** Baseline characteristics of participants by study group and gender

	Total	Males	Females	Intervention vs control school (uncorrected)	Intervention vs control school (corrected)
Age (years)	8.4 ± 2.0 (379)	8.3 ± 2.0 (192)	8.4 ± 1.9 (187)	*t* *= 3.18*	
Intervention school	8.1 ± 2.0 (252)	8.2 ± 2.0 (129)	8.0 ± 1.9 (123)	*SMD = 0.346*	
Control school	8.8 ± 1.8 (127)	8.6 ± 1.8 (63)	9.0 ± 1.8 (64)	*p* *= 0.002*	
Daily MVPA (min)	55 ± 22 (118)	60 ± 20 (56)	50 ± 22 (62)	*t* = 0.766	F = 1.982
Intervention school	53 ± 22 (56)	53 ± 19 (24)	53 ± 24 (32)	SMD = 0.141	SMD = 0.258
Control school	56 ± 22 (62)	66 ± 19 (32)	47 ± 20 (30)	*p* = 0.445	*p* = 0.162
Daily sedentary time (min)	345 ± 74 (118)	338 ± 80 (56)	352 ± 67 (62)	*t* = 1.142	*F = 7.543*
Intervention school	337 ± 77 (56)	327 ± 84 (24)	344 ± 71 (32)	SMD = 0.210	*SMD = 0.493*
Control school	352 ± 71 (62)	346 ± 78 (32)	360 ± 63 (30)	*p* = 0.256	*p* *= 0.007*
Total shuttle distance (m)	670 ± 351 (357)	748 ± 399 (183)	589 ± 271 (174)	*t* = 1.944	F = 0.294
Intervention school	645 ± 351 (240)	719 ± 397 (124)	566 ± 276 (116)	SMD = 0.219	SMD = 0.061
Control school	722 ± 347 (117)	807 ± 400 (59)	635 ± 258 (58)	*p* = 0.053	*p* = 0.588
Sum of skinfolds (mm)	35.1 ± 14.7 (327)	32.6 ± 14.9 (175)	38.1 ± 13.9 (152)	*t* *= 2.214*	F = 2.117
Intervention school	33.8 ± 12.7 (213)	31.7 ± 13.5 (115)	36.4 ± 11.2 (98)	*SMD = 0.257*	SMD = 0.169
Control school	37.6 ± 17.6 (114)	34.4 ± 17.3 (60)	41.1 ± 17.4 (54)	*p* *= 0.028*	*p* = 0.147
% meeting physical activity guidelines	36.4% (118)	46.4% (56)	27.4% (62)	*χ*^*2*^ *= 4.29,* *p* *= 0.038* *OR = 2.251 (1.037–4.884),* *p* *= 0.040*	
Intervention school	26.8% (56)	25.0% (24)	28.1% (32)		
Control school	45.2% (62)	62.5% (32)	26.7% (30)		
% overweight or obese	16.8% (369)	18.6% (188)	14.9% (181)	χ^2^ = 0.01, *p* = 0.922 OR = 1.029 (0.578–1.833), *p* = 0.928	
Intervention school	16.7% (246)	19.0% (126)	14.2% (120)		
Control school	17.1% (123)	17.7% (62)	16.4% (61)		

Values in columns 2–4 are means ± SD (*n* value) or percentage (*n* value). Comparisons are by *t* test or χ^2^ and odds ratio. Corrected values are from ANOVA including correction for age, gender and age*gender. Shuttle distance is given to the nearest metre. Accelerometer minutes are given to the nearest minute. It was not possible to correct percentage values for age and gender. MVPA and sedentary time were also corrected for wear time. This table includes only participants with valid measurements both before and after the intervention

*MVPA* moderate to vigorous intensity physical activity, *OR* odds ratio, *SD* standard deviation, *SMD* standardised mean difference

**Table 2:** Baseline characteristics of participants by study group and SIMD

	Total	SIMD 4–5	SIMD 1–3	SIMD 1–3 vs SIMD 4–5
Age (years)	8.4 ± 2.0 (379)	8.4 ± 1.9 (264)	8.4 ± 2.0 (115)	*t* = 0.033
Intervention school	8.1 ± 2.0 (252)	8.1 ± 2.0 (182)	8.2 ± 2.1 (70)	SMD = 0.004
Control school	8.8 ± 1.8 (127)	8.9 ± 1.8 (82)	8.6 ± 1.8 (45)	*p* = 0.974
Daily MVPA (min)	55 ± 22 (118)	56 ± 21 (92)	51 ± 25 (26)	*t* = 1.024
Intervention school	53 ± 22 (56)	52 ± 19 (42)	57 ± 29 (14)	SMD = 0.227
Control school	56 ± 22 (62)	60 ± 22 (50)	44 ± 17 (12)	*p* = 0.308
Daily sedentary time (min)	345 ± 74 (118)	342 ± 70 (92)	356 ± 86 (26)	*t* = 0.879
Intervention school	337 ± 77 (56)	337 ± 70 (42)	337 ± 97 (14)	SMD = 0.195
Control school	352 ± 71 (62)	346 ± 70 (50)	378 ± 70 (12)	*p* = 0.381
Total shuttle distance (m)	670 ± 351 (357)	702 ± 368 (252)	593 ± 294 (105)	*t* *= 2.695*
Intervention school	645 ± 351 (240)	658 ± 365 (173)	613 ± 314 (67)	*SMD = 0.313*
Control school	722 ± 347 (117)	800 ± 359 (79)	559 ± 255 (38)	*p* *= 0.007*
Sum of skinfolds (mm)	35.1 ± 14.7 (327)	32.9 ± 13.0 (229)	40.4 ± 16.9 (98)	*t* *= 4.350*
Intervention school	33.8 ± 12.7 (213)	32.4 ± 11.9 (154)	37.5 ± 14.0 (59)	*SMD = 0.525*
Control school	37.6 ± 17.6 (114)	33.8 ± 15.0 (75)	44.8 ± 20.0 (39)	*p* *< 0.001*
% meeting physical activity guidelines	36.4% (118)	39.1% (92)	26.9% (26)	*χ*^*2*^ *= 1.304,* *p* *= 0.253* *OR = 0.573 (0.219–1.500),* *p* *= 0.260*
Intervention school	26.8% (56)	26.2% (42)	28.6% (14)	
Control school	45.2% (62)	50.0% (50)	25.0% (12)	
% overweight or obese	16.8% (369)	12.4% (259)	27.3% (110)	*χ*^*2*^ *= 12.291,* *p* *< 0.001* *OR = 2.660 (1.520–4.655),* *p* *< 0.001*
Intervention school	16.7% (246)	12.4% (178)	27.9% (68)	
Control school	17.1% (123)	12.3% (81)	26.2% (42)	

Values in columns 2–4 are means ± SD (*n* value) or percentage (*n* value). Comparisons are by *t* test or χ^2^ and odds ratio. Shuttle distance is given to the nearest metre. Accelerometer minutes are given to the nearest minute. This table includes only participants with valid measurements both before and after the intervention

*MVPA* moderate to vigorous intensity physical activity, *OR* odds ratio, *SD* standard deviation, *SIMD* Scottish Index of Multiple Deprivation, *SMD* standardised mean difference

Consent for a complete set of measurements was given in the majority of cases; although, on the day of testing, some pupils did not wish to give verbal consent for skinfold measurements, had worn inappropriate clothing for some skinfold measurements or were unable to complete individual tests due to pre-existing minor injuries or ailments. In total, at both baseline and follow-up, between 84% and 94% of originally consented participants had data on fitness and body composition outcomes and the proportions were similar at both baseline and follow-up, in the intervention and control schools and in males and females (Fig. 1).

Three pupils refused to wear accelerometers. The proportion of pupils with valid accelerometer data was lower than other measurements although the proportion of males and females was similar. Only 32% of participants in the intervention school and 67% in the control school had valid accelerometer data at baseline whilst at follow-up 65% and 64% respectively had valid accelerometer data (Fig. 1). This was partially because of the requirement that wearers had at least 10 hours of valid wear time on at least 3 days [31]. However, due to a desire to start doing the Daily Mile as soon as possible in the intervention school and a limited number of accelerometers (117), it was possible to collect true baseline data (i.e. prior to beginning the Daily Mile) from only a portion of the intervention school participants. These participants were selected at random based on the availability of pupils for the other physiological tests.

The baseline characteristics of the participants are given in Table 1. Age was significantly higher in the control school at baseline due to the difference in the time of baseline measurements meaning that the pupils were slightly further through the academic year at the control school (Table 1). The percentage meeting the physical activity guidelines of 60+ min MVPA per day and the sum of skinfolds were both higher in the control school. However, after correction for age, gender and age*gender, only sedentary time differed significantly between the schools at baseline, suggesting that the correct confounders were accounted for in the analysis. Additionally, the intra-school differences between genders and year groups were similar (Additional file 1: Table S1).

SIMD scores were similar across schools (χ^2^ = 1.299, *p* = 0.254). For quintiles 4–5 (least deprived), the scores were 71% versus 65%, respectively, for the intervention school and control school. For quintiles 1–3 (most deprived), the scores were 29% versus 35%. These reflect the lower deprivation than the average across Scotland [35], since ~20% would be expected in each quintile. However, this excess of children from less deprived areas reduces the likelihood of observing an impact of the Daily Mile, rather than creating any potential artefacts, as the children are more likely to be fitter and less likely to be overweight or obese [22, 23] at baseline. Nonetheless, children from areas with lower socioeconomic scores (quintiles 1–3) had similar minutes of MVPA and sedentary time compared to those with higher socioeconomic scores (quintiles 4–5). They were also equally likely to meet the physical activity guidelines. However, they had lower shuttle distance, and a higher sum of skinfolds and rates of overweight and obesity (Table 2). A full breakdown of baseline characteristics by socioeconomic group, school and gender is given in Additional file 1: Table S2.

In the main analysis, after adjustment for the common confounders of age, gender and age*gender, significant improvements were observed in the intervention school relative to the control school for MVPA (+9.1 min), sedentary time (−18.2 min), fitness (+39.1 m) and body composition (−1.4 mm; Table 3). These relationships persisted after including a correction for SIMD (Table 3). Baseline values and change in BMI *z* score and age-corrected $\dot{\mathrm{V}}{\mathrm{O}}_2\max$ scores are given in Additional file 1: Table S1–S3 only for comparison with other studies.

**Table 3:** Effect of introducing the Daily Mile on outcomes assessed immediately after the end of the intervention period

Outcome	Difference in change between schools after correction for age, gender and age*gender			Difference in change between schools after correction for age, gender, age*gender and SIMD		
	Mean (95% CI)	SMD	*p* value	Mean (95% CI)	SMD	*p* value
Mean MVPA per day (min)	9.1 (5.1 to 13.2)	0.407	*0.027*	9.5 (5.4 to 13.5)	0.422	*0.021*
Mean sedentary time per day (min)	-18.2 (−10.7 to −25.7)	0.437	*0.017*	-18.1 (−10.6 to − 25.6)	0.435	*0.018*
Total shuttle distance (m)	39.1 (21.9 to 56.3)	0.236	*0.037*	37.2 (20.1 to 54.3)	0.225	*0.046*
Sum of skinfolds (mm)	-1.4 (−2.0 to −0.8)	0.246	*0.034*	-1.4 (−2.0 to − 0.8)	0.258	*0.026*

SMD is calculated as the change in the intervention school relative to the control school as a proportion of the standard deviation of the change. Analyses were conducted using GLM-ANOVA corrected for age, gender, age*gender ± SIMD with repeated measures for the outcome. Sedentary time and MVPA were also corrected for accelerometer wear time

*95% CI* 95% confidence interval, *MVPA* moderate to vigorous intensity physical activity, *SIMD* Scottish Index of Multiple Deprivation, *SMD* standardised mean difference

**Fig. 2:**
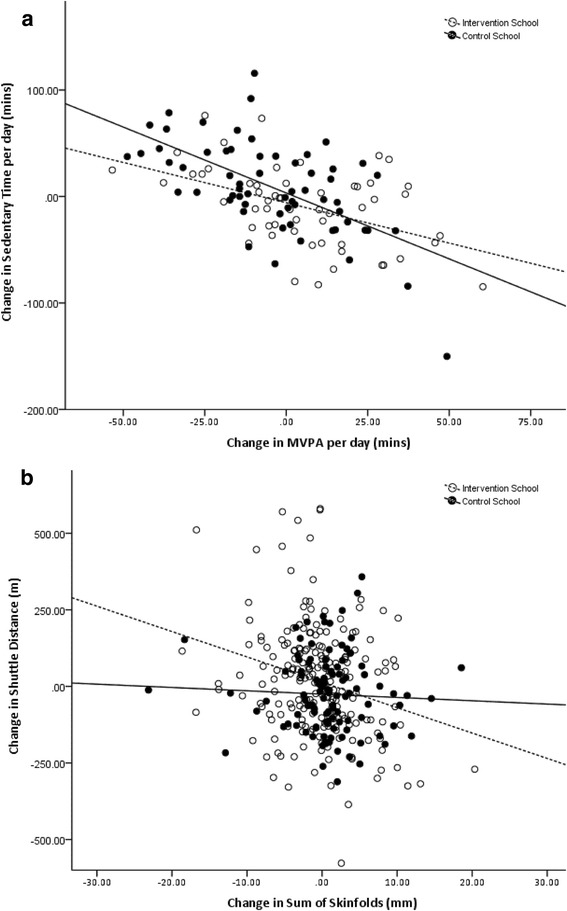
Relationship between change in (**a**) MVPA and sedentary behaviour and (**b**) shuttle distance and sum of skinfolds. Both graphs are drawn after correction for gender, age in days and gender*age in days. MVPA moderate to vigorous intensity physical activity

A change in MVPA predicted a change in sedentary behaviour (*r* = −0.559, *n* = 118, *p* < 0.001; Fig. 2a) but did not predict changes in other primary outcome variables. A change in shuttle distance predicted a change in sum of skinfolds (*r* = −0.203, *n* = 317, *p* < 0.001; Fig. 2b) but did not predict changes in other primary outcome variables. These correlations were not significantly altered by the inclusion of SIMD (*r* = −0.564 and −0.212, respectively) although a change in MVPA additionally predicted a change in shuttle distance (*r* = 0.187, *n* = 115, *p* = 0.046). However, the relationship between a change in sum of skinfolds and a change in shuttle distance differed by school (interaction *p* = 0.043). The relationship was stronger for the intervention school than the control school (*r* = −0.245 and −0.046, respectively). The relationship between a change in MVPA and a change in sedentary behaviour was not different by school (interaction *p* = 0.896). However, it should be noted that the calculations for MVPA and sedentary time are linked by the finite number of minutes in a day and a change in one is likely to result in a change in the other.

## Discussion

In this primary-school-based quasi-experimental pilot study, which investigated the effects of taking part in the Daily Mile, we found evidence of a positive effect on our four primary outcomes—accelerometer-assessed time spent in MVPA, accelerometer-assessed time spent in sedentary behaviour, physical fitness and body composition—after correcting for the common confounders of age and gender with or without socioeconomic grouping.

### Comparisons with other studies

Whilst no other studies have investigated the effect of taking part in the Daily Mile, some have investigated the effect of increasing physical activity throughout the school day or introducing short physical activity breaks into the school day itself [37, 38]. On the whole, they have had mixed results, with some finding alterations in MVPA and others not. This is likely in part due to the different methods used to assess these behaviours, in part due to the different interventions involved and in part due to the different accelerometer cut-points that can be found in the literature. Additionally, some studies use self-reported physical activity measures, which although easier to administer on a large scale, can lead to differing estimates in comparison to accelerometry [39]. Undoubtedly, the age and demographic of the children also has an influence and an intervention that works in one setting may not work in another.

Similarly, some studies have found changes in body composition or fitness whilst others have not [40–42]. The observed effect of the Daily Mile on fitness in the current study may be a result of the type of intervention activity involved (i.e. running) being similar to the fitness test. However, few studies have taken detailed physiological measurements and often assess BMI only. Changes in BMI are observed with some physical activity interventions but mostly in high-BMI groups. The decrease in the sum of skinfolds observed in this study without a concomitant change in BMI *z* score is likely due to the higher resolution of skinfolds and its utility in assessing body fatness without the confounding effect of muscle mass.

### Meaning of study findings

Scottish government figures suggest that 73% of children in Scotland (77% of boys and 69% of girls) meet the physical activity guidelines [14]. However, this figure is based on self-reported questionnaire results rather than accelerometer assessment and is likely to contain bias [43, 44]. Estimates by accelerometer of the percentage of children meeting the physical activity guidelines vary across Europe, from as low as 2% to as high as 63% [11, 45]. The children in this study fall within this range and are likely typical of Scottish and European primary school children [46, 47]. Regardless of how many meet the minimum recommended guidelines (at least 60 min per day), higher levels of MVPA are generally considered to be better. This study shows that introducing the Daily Mile into a primary school setting does increase the MVPA of children by 9.1 min (SMD = 0.407). Although the Daily Mile is a 15-min physical activity intervention, an increase of ~9 min is consistent with the pattern of running interspersed with periods of walking and chatting that is observed in children taking part (personal observations). Although the SMD would be considered to be small according to Cohen [36], small effects on a prevalent behaviour, such as physical inactivity, may have a high impact at the level of population health [48]. In addition, a change of this magnitude is close to the 10 min increase in MVPA previously associated with meaningful reductions in cardiometabolic risk in children and adolescents [49].

Sedentary time is less well studied than physical activity. Nonetheless, the available evidence suggests that the children in this study are typical of European children [11, 45]. In some studies, sedentary time appears to be a predictor of chronic disease independent of physical activity levels [50]. Two aspects of sedentary behaviour appear to be key to this: total sedentary time and prolonged blocks of sedentary time. The Daily Mile is potentially able to address both these issues although the present analysis only investigates total sedentary time. Although children at the intervention school were less sedentary at the baseline (after correction for the common confounders), this would make it harder to observe a reduction in sedentary behaviour rather than easier. Despite this, this study shows an ~18 min reduction (SMD = 0.437) in average daily sedentary time with the introduction of the Daily Mile. Again, this is consistent with a target of 15 min of physical activity since the children will at least be up from their chairs for a slightly longer period. However, if done correctly, the Daily Mile also breaks up the sedentary time, as it should happen in the middle of lessons, so that the children are likely to be sitting before and after their Daily Mile. As for MVPA, the SMD would be considered to be small but may well have significant impacts on population health due to mass participation. Additionally, the data also show a strong correlation between increasing MVPA and reducing sedentary time. This suggests that children are not compensating for the increase in MVPA during the Daily Mile by sitting more at other times of the day: they are replacing sedentary time with MVPA. However, note that the calculations for MVPA and sedentary time are linked by the finite number of minutes in a day and may be more appropriately analysed in future studies using a compositional data analysis.

The children in the IDEFICS study [51] have median values of age-corrected $\dot{\mathrm{V}}{\mathrm{O}}_2\max$ scores between 46.7 and 48.1 ml·kg^− 1^·min^− 1^ for boys and between 45.4 and 47.4 ml·kg^− 1^·min^− 1^ for girls between the ages of 6 and 9 years. Relatively, the children in the current study could be considered to have high aerobic fitness (see Additional file 1: Tables S1 and S2 for age-corrected $\dot{\mathrm{V}}{\mathrm{O}}_2\max$ scores). This high baseline fitness would make it less likely that a change in fitness could be observed after a small increase in physical activity. Nonetheless, an improvement in fitness, as measured by shuttle distance (39.1 m, SMD = 0.236), was observed with the introduction of the Daily Mile. $\dot{\mathrm{V}}{\mathrm{O}}_2\max$ is linked with cardiovascular health and all-cause mortality [52]. Although the SMD would be considered to be small, it may have a significant impact on a population scale. The CARDIA study in young adults suggests that having a $\dot{\mathrm{V}}{\mathrm{O}}_2\max$of 3.5 ml·kg^− 1^·min^− 1^ (approximately 1 metabolic equivalent) higher gives a reduction in all-cause mortality of ~15% [53]. Whilst we only see a relative increase of ~0.35 ml·kg^− 1^·min^− 1^ (Additional file 1: Table S3) with the Daily Mile, this is still predictive of an ~1.5% reduction in all-cause mortality risk. Note that the conversion from shuttle distance to $\dot{\mathrm{V}}{\mathrm{O}}_2\max$ includes age in years and has, therefore, relatively lower resolution. It has also been suggested that having a higher cardiorespiratory fitness at a younger age confers the greatest survival benefit [52]. Furthermore, those with lower starting values appear likely to benefit to a greater extent [54]. This suggests that there are potentially useful health benefits associated with taking part in the Daily Mile.

The children at both schools in this study had lower rates of overweight and obesity than are typical of Scottish children. The Scottish Health Survey reports overweight and obesity rates in 7–11 year olds as 30% (29% for boys and 32% for girls) [14]. Again, this makes it less likely that a change in adiposity could be observed after a small increase in physical activity. Still, a reduction in adiposity as measured by skinfold (1.4 mm, SMD = 0.246) was observed with the introduction of the Daily Mile. Again, although the SMD would be considered to be small, at the population level it may have significant impacts on levels of adiposity. It is also possible that the impact of the Daily Mile on body composition may be larger still in children with higher rates of overweight and obesity. This intervention may be a useful component within measures designed to help tackle the obesity pandemic [8]. The strong correlation between those who reduced their skinfolds the most and those who gained the most fitness may indicate a common cause. Given that the pace each child completes the Daily Mile at is self-determined, it is possible that the children who gained the most benefits took a particular approach to the Daily Mile. An insight into this may come when we interview the children taking part in the Daily Mile about their experiences.

Evidence linking socioeconomic status to MVPA and sedentary behaviour is unclear [13]. This is in part due to the use of different methods of capturing these outcome measures but also due to different ways of assessing socioeconomic status in different countries. Nonetheless, these outcome measures do appear to associate with specific aspects of socioeconomic status in some studies. However, clear differences between higher and lower socioeconomic groupings could be seen in the current study for fitness and body composition: children from postcodes with higher deprivation had lower levels of fitness, higher sums of skinfolds and higher rates of overweight and obesity. This is consistent with the widely recognised health inequality gap [55]. However, no differences were seen between the socioeconomic groupings in response to the introduction of the Daily Mile, suggesting that it may be beneficial to all groups regardless of background. Note that this study was not intended to investigate this, and larger more powerful studies are needed to investigate this aspect of the Daily Mile. A summary of the study and its implications can be found in Box 1.

### Strengths and limitations of study

This study is the first to investigate the widely publicised and adopted Daily Mile physical activity intervention. The intervention appears to be increasingly popular and has now been maintained in the originator school for more than five years. Thus, it is undoubtedly feasible to deliver and has been adopted locally in many areas. What was unknown was the efficacy for the anecdotally reported physiological benefits of taking part in the Daily Mile. Consent rates were high (>77% in both schools) as were the number of children successfully assessed at both time points for most outcome measures. We assessed MVPA and sedentary time using the gold standard accelerometer technique, we assessed fitness using the bleep test (which has been validated in this age group) and we assessed body composition using labour-intensive skinfold assessments rather than the more straightforward but lower resolution BMI.

We acknowledge that there was a difference in sedentary time between the schools at baseline and the socioeconomic groupings were not reflective of the whole of Scotland, which are limitations of our study. However, these differences would be predicted to make any effects of the Daily Mile harder to observe, not easier. Changes were observed despite these differences. It would have been preferable to assess both the intervention and control schools at the same time of year to avoid any seasonal impact on physical activity. However, we believe that October and March should be similar enough to allow comparison [56]. Additionally, it would have been better to have had both schools involved in the study for the same length of time, although, correcting for age and gender should account for this difference. It is also possible that differences in the health and well-being policies within the schools contributed to differences in the results. However, the schools were selected to be from the same local authority and to be of similar socioeconomic make-up to minimise potential differences. As predicted, changes in outcome variables had effect sizes at the smaller end of the distribution (0.2–0.5). However, given the involvement of whole classes, small effects could have an important impact on population health. To gain further confidence in the results, this study should be replicated in a larger number of schools. Furthermore, no monitoring of adherence, or level of adherence, to the intervention was carried out, although the results suggest adherence was sufficient.

### Unanswered questions and future research

Additional anecdotally reported benefits to the Daily Mile (cognition, behaviour and well-being) are currently being investigated [57, 58]. It is essential that the current studies are replicated in a larger number of schools and countries to ensure that the findings are both robust and repeatable in different educational contexts. Future studies should include diet and sleep quality, which we are not yet investigating, to explore the potential mechanisms of impact. More attention should be given to when the Daily Mile is being done during the school day and whether it is breaking up sedentary time. Additionally, future studies should investigate whether MVPA and sedentary behaviour are changing on weekdays and/or weekend days.

In 2015, the Scottish government launched the Scottish Attainment Challenge with the aim of achieving equity in educational outcomes for all Scottish children [59]. Whilst the current study found no difference in the response to the Daily Mile by socioeconomic grouping, both schools were heavily weighted towards less deprived catchment areas. Furthermore, the current study was not powered to detect such a complex interaction. The Daily Mile is a free, simple intervention that can be rolled out to schools regardless of socioeconomic status. It is necessary to conduct carefully designed studies to understand the impact of the Daily Mile in different socioeconomic settings and to understand whether it can have any impact on the attainment gap.

The sample of children participating in this study included a number with challenging behaviours including autism spectrum disorders. Nonetheless, they took part in the Daily Mile and our investigations. Understanding the impact of the Daily Mile on children with differing learning needs should also be a future priority.

This study shows the value of introducing the Daily Mile into schools. Whilst the Daily Mile has been introduced as policy across Scotland, many schools do not have appropriate outdoor facilities to allow their children to take part. One of the challenges for policymakers and other stakeholders is to consider how to introduce the Daily Mile or alternative interventions that have been shown to increase MVPA and fitness into such schools or how to adapt those schools to allow the introduction of appropriate interventions.

## Conclusions

In conclusion, introducing the Daily Mile to the primary school day appears to be an effective intervention for increasing MVPA and reducing sedentary time and it has measurable impacts on key aspects of metabolic health: body composition and physical fitness. This study provides the first assessment of the Daily Mile and it will allow the development of evidence-based policy around introducing the Daily Mile to more schools.

## Box 1: What this study adds

Why was this study done?

- Low physical activity, high sedentary behaviour, declining fitness levels and high levels of overweight and obesity are global problems that have been targeted by the World Health Organisation.
- The Daily Mile is an increasingly popular school-based physical activity intervention, backed by the Scottish government, which is anecdotally reported to lead to increased physical activity, reduced sedentary time, improved fitness and improved body composition. Pupils run or walk laps of the playground at a self-selected pace for 15 min during normal classroom time. It is increasingly popular throughout the UK, in parts of Europe and some schools in the USA.
- However, these reported benefits remain anecdotal and need to be quantitatively and objectively assessed to ensure that the loss of academic classroom time is providing the reported alternative benefits.

What did the researchers do and find?

- Two schools in the Stirling Council area, Scotland, were recruited: one with intention to start the Daily Mile, the other without.
- Researchers assessed the physical activity and sedentary behaviour of children using accelerometers, their fitness using the bleep test and their body composition using skinfolds. This was done in both schools before and after the intervention school introduced the Daily Mile.
- This quasi-experimental pilot study found that, after correcting for age, gender and socioeconomic grouping, taking part in the Daily Mile did lead to an improvement in physical activity, sedentary behaviour, fitness and body composition of children in the intervention school relative to the control school.

What do these findings mean?

- This suggests that the Daily Mile is a worthwhile intervention to introduce in schools and that it should be considered for inclusion in government policy.
- This study can underpin the policy already introduced by the Scottish government and the development of future policy in other parts of the UK and abroad.

## Additional file

Additional file 1: **Table S1.** Baseline characteristics of participants by year group and study group. **Table S2.** Baseline characteristics of participants by socioeconomic group, school and gender. **Table S3.** Effect of introducing the Daily Mile on additional outcomes assessed immediately after the end of the intervention period for comparison with other studies. (DOCX 65 kb)

## Abbreviations

95%CI: 95% confidence interval
BMI: Body mass index
cpm: Counts per minute
ISAK: International Society for the Advancement of Kinanthropometry
MVPA: Moderate to vigorous intensity physical activity
PE: Physical education
SIMD: Scottish Index of Multiple Deprivation
SMD: Standardised mean difference
WHEEL: Well-being, Health, Exercise, Enjoyment and Learning
WHO: World Health Organisation
