# Supplementary material for: The Daily Mile makes primary school children more active, less sedentary and improves their fitness and body composition: a quasi-experimental pilot study
Source: BMC Med. 2018 May 10;16:64. doi: 10.1186/s12916-018-1049-z (PMC5944120; doi:10.1186/s12916-018-1049-z)
Supplement: Supplementary file 1 — Table S1. Baseline characteristics of participants by year group and study group. Table S2. Baseline characteristics of participants by socioeconomic group, school and gender. Table S3. Effect of introducing the Daily Mile on additional outcomes assessed immediately after the end of the intervention period for comparison with other studies. (DOCX 65 kb) [file 12916_2018_1049_MOESM1_ESM.docx]

**Table S1** Baseline characteristics of participants by year group and study group.

|  | **Year Group** | **Intervention School** | | | **Control School** | | |
| --- | --- | --- | --- | --- | --- | --- | --- |
|  |  | **Total** | **Males** | **Females** | **Total** | **Males** | **Females** |
| Age (years) | P1 | 5.2 ± 0.3 (34) | 5.2 ± 0.3 (15) | 5.2 ± 0.3 (19) | 5.7 ± 0.3 (11) | 5.9 ± 0.1 (7) | 5.4 ± 0.2 (4) |
|  | P2 | 6.2 ± 0.3 (40) | 6.2 ± 0.4 (25) | 6.1 ± 0.3 (15) | 6.7 ± 0.3 (17) | 6.7 ± 0.3 (10) | 6.6 ± 0.3 (7) |
|  | P3 | 7.3 ± 0.3 (39) | 7.3 ± 0.3 (17) | 7.2 ± 0.3 (22) | 7.6 ± 0.4 (20) | 7.7 ± 0.4 (11) | 7.5 ± 0.2 (9) |
|  | P4 | 8.3 ± 0.2 (37) | 8.3 ± 0.2 (20) | 8.3 ± 0.2 (17) | 8.7 ± 0.3 (18) | 8.6 ± 0.3 (8) | 8.7 ± 0.3 (10) |
|  | P5 | 9.2 ± 0.3 (34) | 9.2 ± 0.3 (16) | 9.2 ± 0.3 (18) | 9.6 ± 0.3 (27) | 9.6 ± 0.3 (12) | 9.6 ± 0.3 (15) |
|  | P6 | 10.2 ± 0.4 (36) | 10.3 ± 0.3 (17) | 10.1 ± 0.4 (19) | 10.7 ± 0.3 (23) | 10.7 ± 0.3 (11) | 10.7 ± 0.3 (12) |
|  | P7 | 11.2 ± 0.3 (32) | 11.3 ± 0.3 (19) | 11.0 ± 0.3 (13) | 11.7 ± 0.3 (11) | 11.8 ± 0.4 (4) | 11.7 ± 0.3 (7) |
| Average MVPA per day (mins) | P1 | 54 ± 13 (5) | 43 ± 2 (2) | 62 ± 11 (3) | NR (1) | NR (1) | NA (0) |
|  | P2 | 55 ± 22 (18) | 49 ± 16 (11) | 64 ± 28 (7) | 70 ± 20 (8) | 69 ± 23 (6) | 74 ± 4 (2) |
|  | P3 | 56 ± 33 (9) | 72 ± 42 (2) | 52 ± 32 (7) | 62 ± 21 (10) | 71 ± 15 (4) | 57 ± 24 (6) |
|  | P4 | 54 ± 8 (6) | 63 ± 7 (2) | 50 ± 3 (4) | 56 ± 22 (12) | 74 ± 14 (6) | 38 ± 11 (6) |
|  | P5 | 52 ± 25 (13) | 62 ± 34 (3) | 49 ± 23 (10) | 54 ± 21 (12) | 64 ± 18 (7) | 40 ± 17 (5) |
|  | P6 | 37 ± 13 (3) | 44 ± 9 (2) | NR (1) | 45 ± 21 (14) | 52 ± 22 (6) | 41 ± 21 (8) |
|  | P7 | 52 ± 9 (2) | 52 ± 9 (2) | NA (0) | 62 ± 21 (5) | 78 ± 5 (2) | 52 ± 22 (3) |
| Sedentary time per day (mins) | P1 | 267 ± 39 (5) | 293 ± 57 (2) | 249 ± 16 (3) | NR (1) | NR (1) | NA (0) |
|  | P2 | 281 ± 38 (18) | 275 ± 31 (11) | 291 ± 49 (7) | 280 ± 71 (8) | 258 ± 68 (6) | 346 ± 28 (2) |
|  | P3 | 332 ± 72 (9) | 271 ± 25 (2) | 350 ± 72 (7) | 304 ± 47 (10) | 322 ± 58 (4) | 292 ± 38 (6) |
|  | P4 | 333 ± 20 (6) | 323 ± 25 (2) | 338 ± 18 (4) | 343 ± 38 (12) | 343 ± 34 (6) | 343 ± 45 (6) |
|  | P5 | 410 ± 62 (13) | 440 ± 98 (3) | 401 ± 52 (10) | 402 ± 68 (12) | 409 ± 72 (7) | 392 ± 68 (5) |
|  | P6 | 405 ± 32 (3) | 397 ± 41 (2) | NR (1) | 386 ± 64 (14) | 372 ± 64 (6) | 397 ± 66 (8) |
|  | P7 | 468 ± 29 (2) | 468 ± 29 (2) | NA (0) | 393 ± 29 (5) | 405 ± 50 (2) | 385 ± 12 (3) |
| Total Shuttle Distance (m) | P1 | 269 ± 135 (32) | 280 ± 161 (13) | 261 ± 119 (19) | 376 ± 106 (10) | 393 ± 106 (6) | 350 ± 116 (4) |
|  | P2 | 404 ± 157 (36) | 452 ± 164 (24) | 310 ± 87 (12) | 439 ± 196 (16) | 432 ± 234 (10) | 450 ± 127 (6) |
|  | P3 | 572 ± 194 (38) | 606 ± 193 (17) | 545 ± 196 (21) | 653 ± 280 (18) | 784 ± 274 (10) | 490 ± 200 (8) |
|  | P4 | 639 ± 238 (35) | 682 ± 246 (20) | 583 ± 222 (15) | 776 ± 234 (17) | 820 ± 211 (7) | 746 ± 255 (10) |
|  | P5 | 765 ± 281 (33) | 823 ± 265 (15) | 718 ± 293 (18) | 808 ± 344 (26) | 942 ± 389 (12) | 693 ± 261 (14) |
|  | P6 | 894 ± 344 (36) | 1074 ± 373 (17) | 733 ± 220 (19) | 863 ± 374 (23) | 1080 ± 378 (11) | 665 ± 245 (12) |
|  | P7 | 1004 ± 403 (30) | 1120 ± 474 (18) | 830 ± 159 (12) | 1123 ± 329 (7) | 1393 ± 316 (3) | 920 ± 148 (4) |
| Sum of skinfolds (mm) | P1 | 30.3 ± 7.8 (21) | 27.2 ± 4.9 (10) | 33.1 ± 9.1 (11) | 33.6 ± 15.7 (7) | 33.8 ± 17.2 (6) | NR (1) |
|  | P2 | 28.3 ± 8.4 (34) | 24.9 ± 6.7 (22) | 34.5 ± 7.8 (12) | 34.5 ± 17.0 (16) | 36.1 ± 20.6 (10) | 31.8 ± 9.4 (6) |
|  | P3 | 30.4 ± 7.0 (36) | 29.0 ± 8.0 (16) | 31.6 ± 6.1 (20) | 31.4 ± 11.0 (17) | 24.9 ± 6.2 (9) | 38.7 ± 11.0 (8) |
|  | P4 | 31.5 ± 12.3 (32) | 27.2 ± 8.1 (17) | 36.4 ± 14.6 (15) | 33.2 ± 14.7 (17) | 29.0 ± 9.8 (8) | 37.0 ± 17.7 (9) |
|  | P5 | 32.2 ± 10.8 (28) | 28.3 ± 7.2 (14) | 36.0 ± 12.6 (14) | 41.5 ± 18.3 (25) | 35.1 ± 14.5 (12) | 47.4 ± 20.0 (13) |
|  | P6 | 40.5 ± 14.0 (31) | 42.1 ± 17.7 (17) | 38.5 ± 7.5 (14) | 46.9 ± 22.4 (22) | 47.5 ± 24.0 (11) | 46.4 ± 21.8 (11) |
|  | P7 | 43.5 ± 17.3 (31) | 41.2 ± 19.2 (19) | 47.2 ± 13.6 (12) | 33.0 ± 12.8 (10) | 25.7 ± 2.7 (4) | 37.9 ± 14.8 (6) |
| Percent meeting 60+ mins per day activity guidelines (%) | P1 | 40.0% (5) | 00.0% (2) | 66.7% (3) | NR (1) | NR (1) | NA (0) |
|  | P2 | 27.8% (18) | 27.3% (11) | 28.6% (7) | 75.0% (8) | 66.7% (6) | 100.0% (2) |
|  | P3 | 44.4% (9) | 50.0% (2) | 42.9% (7) | 50.0% (10) | 75.0% (4) | 33.3% (6) |
|  | P4 | 16.7% (6) | 50.0% (2) | 00.0% (4) | 41.7% (12) | 83.3% (6) | 00.0% (6) |
|  | P5 | 23.1% (13) | 33.3% (3) | 20.0% (10) | 41.7% (12) | 57.1% (7) | 20.0% (5) |
|  | P6 | 00.0% (3) | 00.0% (2) | NR (1) | 28.6% (14) | 33.3% (6) | 25.0% (8) |
|  | P7 | 00.0% (2) | 00.0% (2) | NA (0) | 60.0% (5) | 100.0% (2) | 33.3% (3) |
| Percent overweight or obese (%) | P1 | 21.4% (28) | 23.1% (13) | 20.0% (15) | 21.7% (23) | 22.2% (9) | 21.4% (14) |
|  | P2 | 31.0% (29) | 26.7% (15) | 35.7% (14) | 25.0% (20) | 10.0% (10) | 40.0% (10) |
|  | P3 | 16.1% (31) | 16.7% (18) | 15.4% (13) | 17.4% (23) | 23.1% (13) | 10.0% (10) |
|  | P4 | 37.5% (24) | 23.1% (13) | 54.5% (11) | 35.3% (17) | 18.2% (11) | 66.7% (6) |
|  | P5 | 32.1% (28) | 23.1% (13) | 40.0% (15) | 25.0% (16) | 12.5% (8) | 37.5% (8) |
|  | P6 | 39.5% (38) | 50.0% (22) | 25.0% (16) | 34.8% (23) | 53.8% (13) | 10.0% (10) |
|  | P7 | 47.4% (19) | 61.5% (13) | 16.7% (6) | 56.3% (16) | 66.7% (12) | 25.0% (4) |
| $\dot{V}O_{2}max$ (ml∙kg^-1^∙min^-1^) | P1 | 52.1 ± 1.6 (32) | 52.6 ± 1.7 (13) | 51.8 ± 1.5 (19) | 52.7 ± 1.1 (10) | 52.6 ± 0.7 (6) | 52.8 ± 1.6 (4) |
|  | P2 | 51.7 ± 2.0 (36) | 52.2 ± 2.2 (24) | 50.8 ± 1.3 (12) | 51.5 ± 2.5 (16) | 51.3 ± 2.8 (10) | 51.8 ± 2.1 (6) |
|  | P3 | 51.9 ± 2.4 (38) | 52.3 ± 2.3 (17) | 51.6 ± 2.5 (21) | 52.0 ± 3.1 (18) | 53.2 ± 3.0 (10) | 50.6 ± 2.8 (8) |
|  | P4 | 50.9 ± 3.2 (35) | 51.4 ± 3.1 (20) | 50.2 ± 3.3 (15) | 52.2 ± 2.7 (17) | 52.7 ± 2.6 (7) | 51.8 ± 2.8 (10) |
|  | P5 | 51.0 ± 3.2 (33) | 51.7 ± 3.1 (15) | 50.5 ± 3.3 (18) | 50.9 ± 3.9 (26) | 52.5 ± 3.9 (12) | 49.6 ± 3.4 (14) |
|  | P6 | 51.2 ± 4.1 (36) | 53.2 ± 4.2 (17) | 49.5 ± 3.1 (19) | 49.7 ± 4.5 (23) | 52.3 ± 4.5 (11) | 47.4 ± 3.2 (12) |
|  | P7 | 50.9 ± 4.8 (30) | 52.0 ± 5.6 (18) | 49.1 ± 2.4 (12) | 52.0 ± 4.0 (7) | 55.3 ± 3.8 (3) | 49.5 ± 2.0 (4) |
| BMI z-score | P1 | 0.298 ± 0.721 (33) | 0.279 ± 0.692 (14) | 0.312 ± 0.761 (19) | 0.011 ± 1.142 (10) | 0.311 ± 1.254 (6) | -0.437 ± 0.918 (4) |
|  | P2 | 0.123 ± 0.828 (37) | -0.076 ± 0.715 (24) | 0.491 ± 0.924 (13) | 0.138 ± 1.299 (17) | 0.236 ± 1.593 (10) | -0.003 ± 0.809 (7) |
|  | P3 | 0.239 ± 0.701 (39) | 0.343 ± 0.827 (17) | 0.158 ± 0.593 (22) | 0.006 ± 0.641 (20) | 0.005 ± 0.572 (11) | 0.008 ± 0.753 (9) |
|  | P4 | -0.045 ± 0.950 (37) | -0.059 ± 0.983 (20) | -0.029 ± 0.940 (17) | 0.033 ± 0.960 (18) | -0.120 ± 1.026 (8) | 0.155 ± 0.941 (10) |
|  | P5 | 0.072 ± 0.956 (33) | 0.029 ± 0.808 (15) | 0.109 ± 1.086 (18) | 0.139 ± 0.970 (26) | 0.091 ± 0.783 (12) | 0.180 ± 1.134 (14) |
|  | P6 | 0.297 ± 0.889 (36) | 0.649 ± 0.879 (17) | -0.019 ± 0.791 (19) | 0.465 ± 1.278 (22) | 0.808 ± 1.135 (11) | 0.121 ± 1.372 (11) |
|  | P7 | 0.298 ± 0.952 (31) | 0.478 ± 1.032 (19) | 0.013 ± 0.765 (12) | -0.557 ± 0.906 (10) | -0.572 ± 0.652 (4) | -0.547 ± 1.106 (6) |

Values are means ± SD (n-value). MVPA is moderate to vigorous physical activity. Shuttle distance is given to nearest metre. Accelerometer minutes are given to nearest minute. NA = no individuals in that category. NR = not reported to prevent association of individual with their score. Note that this table only includes participants with valid measurements both before and after the intervention.

**Table S2** Baseline characteristics of participants by socioeconomic group, school and gender.

|  | **Intervention School** | | | **Control School** | | |
| --- | --- | --- | --- | --- | --- | --- |
|  | **Total** | **Males** | **Females** | **Total** | **Males** | **Females** |
| Age (years) | 8.1 ± 2.0 (252) | 8.2 ± 2.0 (129) | 8.0 ± 1.9 (123) | 8.8 ± 1.8 (127) | 8.6 ± 1.8 (63) | 9.0 ± 1.8 (64) |
| *SIMD 4-5* | 8.1 ± 2.0 (182) | 8.3 ± 2.0 (91) | 7.9 ± 1.9 (91) | 8.9 ± 1.8 (82) | 8.9 ± 1.7 (43) | 8.9 ± 1.9 (39) |
| *SIMD 1-3* | 8.2 ± 2.1 (70) | 8.1 ± 2.2 (38) | 8.4 ± 1.9 (32) | 8.6 ± 1.8 (45) | 7.9 ± 1.8 (20) | 9.1 ± 1.7 (25) |
| Daily MVPA (mins) | 53 ± 22 (56) | 53 ± 19 (24) | 53 ± 24 (32) | 56 ± 22 (62) | 66 ± 19 (32) | 47 ± 20 (30) |
| *SIMD 4-5* | 52 ± 19 (42) | 54 ± 19 (15) | 51 ± 20 (27) | 60 ± 22 (50) | 70 ± 17 (28) | 47 ± 21 (22) |
| *SIMD 1-3* | 57 ± 29 (14) | 52 ± 22 (9) | 68 ± 41 (5) | 44 ± 17 (12) | 39 ± 12 (4) | 46 ± 20 (8) |
| Daily Sedentary time (mins) | 337 ± 77 (56) | 327 ± 84 (24) | 344 ± 71 (32) | 352 ± 71 (62) | 346 ± 78 (32) | 360 ± 63 (30) |
| *SIMD 4-5* | 337 ± 70 (42) | 319 ± 71 (15) | 347 ± 69 (27) | 346 ± 70 (50) | 338 ± 75 (28) | 356 ± 64 (22) |
| *SIMD 1-3* | 337 ± 97 (14) | 341 ± 105 (9) | 330 ± 90 (5) | 378 ± 70 (12) | 396 ± 86 (4) | 369 ± 65 (8) |
| Total Shuttle Distance (m) | 645 ± 351 (240) | 719 ± 397 (124) | 566 ± 276 (116) | 722 ± 347 (117) | 807 ± 400 (59) | 635 ± 258 (58) |
| *SIMD 4-5* | 658 ± 365 (173) | 747 ± 407 (88) | 565 ± 290 (85) | 800 ± 359 (79) | 923 ± 406 (41) | 667 ± 242 (38) |
| *SIMD 1-3* | 613 ± 314 (67) | 651 ± 367 (36) | 569 ± 237 (31) | 559 ± 255 (38) | 542 ± 227 (18) | 574 ± 283 (20) |
| Sum of skinfolds (mm) | 33.8 ± 12.7 (213) | 31.7 ± 13.5 (115) | 36.4 ± 11.2 (98) | 37.6 ± 17.6 (114) | 34.4 ± 17.3 (60) | 41.1 ± 17.4 (54) |
| *SIMD 4-5* | 32.4 ± 11.9 (154) | 30.6 ± 13.4 (82) | 34.5 ± 9.6 (72) | 33.8 ± 15.0 (75) | 28.6 ± 10.5 (42) | 40.5 ± 17.3 (33) |
| *SIMD 1-3* | 37.5 ± 14.0 (59) | 34.3 ± 13.7 (33) | 41.6 ± 13.5 (26) | 44.8 ± 20.0 (39) | 47.9 ± 22.3 (18) | 42.1 ± 18.0 (21) |
| % meeting PA guidelines | 26.8% (56) | 25.0% (24) | 28.1% (32) | 45.2% (62) | 62.5% (32) | 26.7% (30) |
| *SIMD 4-5* | 26.2% (42) | 26.7% (15) | 25.9% (27) | 50.0% (50) | 71.4% (28) | 22.7% (22) |
| *SIMD 1-3* | 28.6% (14) | 22.2% (9) | 40.0% (5) | 25.0% (12) | 00.0% (4) | 37.5% (8) |
| % overweight or obese | 16.7% (246) | 19.0% (126) | 14.2% (120) | 17.1% (123) | 17.7% (62) | 16.4% (61) |
| *SIMD 4-5* | 12.4% (178) | 14.6% (89) | 10.1% (89) | 12.3% (81) | 9.3% (43) | 15.8% (38) |
| *SIMD 1-3* | 27.9% (68) | 29.7% (37) | 25.8% (31) | 26.2% (42) | 36.8% (19) | 17.4% (23) |
| $\dot{V}O_{2}max$ (ml∙kg^-1^∙min^-1^) | 51.4 ± 3.2 (240) | 52.2 ± 3.4 (124) | 50.6 ± 2.8 (116) | 51.3 ± 3.5 (117) | 52.6 ± 3.3 (59) | 50.1 ± 3.3 (58) |
| *SIMD 4-5* | 51.6 ± 3.1 (173) | 52.5 ± 3.2 (88) | 50.8 ± 2.7 (85) | 52.1 ± 3.4 (79) | 53.3 ± 3.4 (41) | 50.7 ± 3.0 (38) |
| *SIMD 1-3* | 50.8 ± 3.4 (67) | 51.5 ± 3.7 (36) | 50.0 ± 2.7 (31) | 49.8 ± 3.1 (38) | 50.8 ± 2.4 (18) | 48.8 ± 3.5 (20) |
| BMI z-score | 0.180 ± 0.858 (246) | 0.217 ± 0.880 (126) | 0.142 ± 0.837 (120) | 0.093 ± 1.052 (123) | 0.178 ± 1.072 (62) | 0.007 ± 1.033 (61) |
| *SIMD 4-5* | 0.054 ± 0.857 (178) | 0.091 ± 0.928 (89) | 0.017 ± 0.784 (89) | -0.028 ± 0.929 (81) | -0.067 ± 0.826 (43) | 0.015 ± 1.043 (38) |
| *SIMD 1-3* | 0.511 ± 0.774 (68) | 0.519 ± 0.674 (37) | 0.502 ± 0.890 (31) | 0.327 ± 1.235 (42) | 0.730 ± 1.356 (19) | -0.006 ± 1.040 (23) |

Values are means ± SD. MVPA is moderate to vigorous physical activity. After correction for gender and age at time of testing, no significant differences were observed between the schools. Shuttle distance is given to nearest metre. Accelerometer minutes are given to nearest minute. NA = no individuals in that category. Note that this table only includes participants with valid measurements both before and after the intervention.

**Table S3** Analyses to investigate the effect of introducing the Daily Mile on additional outcomes assessed immediately after the end of the intervention period for comparison with other studies.

| **Outcome** | **Difference in change between schools after correction for age, gender and age*gender** | | | **Difference in change between schools after correction for age, gender, age*gender and SIMD** | | |
| --- | --- | --- | --- | --- | --- | --- |
|  | **Mean (95%CI)** | **SMD** | **p-value** | **Mean (95%CI)** | **SMD** | **p-value** |
| $\dot{V}O_{2}max$ (ml∙kg^-1^∙min^-1^) | 0.35 (0.10 to 0.59) | 0.145 | 0.196 | 0.31 (0.07 to 0.55) | 0.132 | 0.243 |
| BMI z-score | -0.008 (-0.035 to 0.019) | 0.030 | 0.785 | -0.008 (-0.035 to 0.019) | 0.030 | 0.784 |

SMD is standardised mean differences. SMD are calculated as change in intervention school relative to the control school as a proportion of the standard deviation of the change. 95%CI is 95% confidence interval. Analyses were conducted using GLM-ANOVA corrected for age, gender, age*gender ± SIMD with repeated measures for the outcome.
